# Supplementary material for: Interventricular Septum Thickness for the Prediction of Coronary Heart Disease and Myocardial Infarction in Hypertension Population: A Prospective Study
Source: J Clin Med. 2022 Dec 1;11(23):7152. doi: 10.3390/jcm11237152 (PMC9738248; doi:10.3390/jcm11237152)
Supplement: Supplementary file 1 [file jcm-2025784-supplementary.pdf › jcm-2025784-supplementary.pdf]

**Supplemental Table S1.** IVS hypertrophy for the prediction of CHD, MI and stroke during follow-up after excluding the kidney disease people

| Subgroups | CHD | | | MI | | |
|---|---|---|---|---|---|---|
| | Cases /Noncases | Incidence HR (95% CI) | P Value | Cases /Noncases | Incidence HR (95% CI) | P Value |
| *Before Adjustment* | | | | | | |
| Overall | | | | | | |
| Normal IVSd | 120/4295 | Reference | / | 51/4364 | Reference | / |
| IVS hypertrophy | 26/356 | 2.755(1.800-4.216) | < 0.001 | 16/366 | 4.154(2.356-7.327) | < 0.001 |
| Male | | | | | | |
| Normal IVSd | 46/2128 | Reference | / | 22/2152 | Reference | / |
| IVS hypertrophy | 9/148 | 2.890(1.413-5.913) | 0.004 | 4/153 | 2.730(0.937-7.953) | 0.066 |
| Female | | | | | | |
| Normal IVSd | 74/2167 | Reference | / | 29/2212 | Reference | / |
| IVS hypertrophy | 17/208 | 2.557(1.506-4.341) | 0.001 | 12/213 | 4.948(2.496-9.811) | < 0.001 |
| *After Adjustment* | | | | | | |
| Overall | | | | | | |
| Normal IVSd | 120/4295 | Reference | / | 51/4364 | Reference | / |
| IVS hypertrophy | 26/356 | 1.818(1.155-2.861) | 0.010 | 16/366 | 2.410 (1.303-4.458) | 0.005 |
| Male | | | | | | |
| Normal IVSd | 46/2128 | Reference | / | 22/2152 | Reference | / |
| IVS hypertrophy | 9/148 | 1.873 (0.875-4.008) | 0.106 | 4/153 | 1.639 (0.516-5.208) | 0.402 |
| Female | | | | | | |
| Normal IVSd | 74/2167 | Reference | / | 29/2212 | Reference | / |
| IVS hypertrophy | 17/208 | 1.870 (1.062-3.292) | 0.030 | 12/213 | 3.063 (1.449-6.473) | 0.003 |

Adjusted for Sex, Age, DM, BMI, Current_smoke, Current_drink, FamilyHis, Mean_SBP, Mean_DBP, GLU, LDL_C, HDL_C as appropriate.
